# Programmed cell death in cancer: targeting necroptosis to kill tumor cell

**DOI:** 10.1038/s41420-026-03002-4

**Published:** 2026-04-06

**Authors:** Jiahao Liang, Chenchen Tan, Xia Li, Jialong Fan, Bin Liu

**Affiliations:** 1 Qingdao Municipal Hospital (Qingdao Hospital, University of Health and Rehabilitation Sciences), Qingdao, China; 2 College of Biology, Hunan University, Changsha, China; 3 Hunan Provincial Key Laboratory of the Research and Development of Novel Pharmaceutical Preparations, Changsha Medical College, Changsha, China; 4 Department of Pharmacy, Qingdao Eye Hospital of Shandong First Medical University, Qingdao, China

**Keywords:** Cancer therapy, Cell death

## Abstract

Necroptosis is a precisely regulated form of programmed cell death (PCD) that exhibits necrotic morphology while being orchestrated receptor-interacting protein kinase 1 (RIPK1), receptor-interacting protein kinase 3 (RIPK3), and mixed lineage kinase domain-like pseudokinase (MLKL). In tumor biology, necroptosis plays a context-dependent dual role: it can suppress tumor progression by inducing immunogenic cell death (ICD) and activating anti-tumor immune responses; yet it may also promote tumor progression and immunosuppression by triggering inflammatory responses. Emerging evidence indicates that small molecule compounds, natural products, and nanomedicine technologies can effectively induce necroptosis in tumor cells, providing opportunities to overcome traditional chemotherapy resistance and enhance anti-tumor immunity. However, clinical translation faces numerous challenges, including frequent downregulation of key necroptotic proteins, the lack of robust predictive biomarkers, and potential tumor-promoting effects. This review offers an integrative perspective linking necroptosis molecular mechanisms, dual functional outcomes, and therapeutic strategies, highlighting both opportunities and risks. By providing mechanistic insights and a framework for rational design of necroptosis-based interventions, this work aims to guide future research toward effective and safe anticancer therapies.

**Figure:**
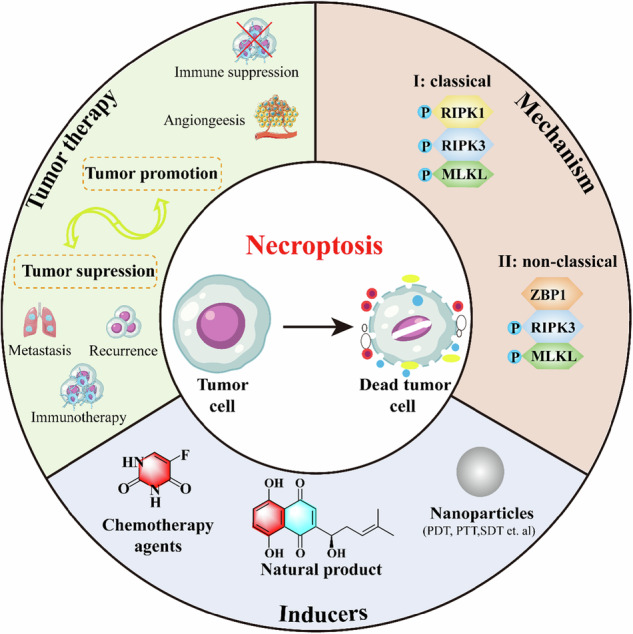
Schematic illustration of the mechanisms, dual roles in tumor therapy, and inducers of necroptosis.

Schematic illustration of the mechanisms, dual roles in tumor therapy, and inducers of necroptosis.

## Facts

- Necroptosis exhibits context-dependent anti- or pro-tumor effects requiring careful evaluation in different tumor types.
- Key necroptosis proteins are often deficient in tumors, limiting therapeutic efficacy.
- Therapeutic induction of necroptosis carries potential risks of promoting tumor progression if not carefully controlled.
- Developing targeted necroptosis inducers represents a promising to overcome apoptosis resistance and enhance antitumor immunity.

## Introduction

Cancer represents a significant public health concern on a global scale and is among the leading diseases threatening population health worldwide [1]. With an aging population, effective tumor treatment, prevention, and management are expected to face increasing challenges [2]. Cancer cell death may occur during development or in response to stressors, such as metabolic disturbances, infection, or tissue damage [3]. In certain instances, cell death results from overwhelming acute events, such as physiological injury or accidental cell death [4]. Conversely, cell death can proceed through regulated specific molecular pathways, collectively termed programmed cell death (PCD), which are amenable to pharmacological or genetic modulation PCD [5]. This process is orchestrated by signal amplification complexes that govern cell fate and influence the tumor immune response [6].

Necroptosis, first described by Degterev et al. [7], is a form of PCD exhibiting necrotic morphology while being mechanistically regulated by RIPK1, RIPK3, and MLKL [8, 9]. The classical necroptosis is triggered by tumor necrosis factor-alpha (TNF-α) binding to tumor necrosis factor receptor 1 (TNFR1), forming of complex I [10]. When caspase-8 activity is inhibited, RIPK1 dissociates from complex I and recruits RIPK3 to form the necrosome (complex II), resulting in RIPK3-mediated phosphorylation of MLKL [11, 12]. Phosphorylated MLKL oligomerizes and translocates to the plasma membrane, causing membrane disruption and cell death [13]. Beyond the TNF-α pathway, Z-DNA binding protein 1 (ZBP1) can initiate necroptosis independently of RIPK1 in response to viral infection or metabolic stress such as glucose deprivation [14].

In cancer biology, necroptosis exhibits a context-dependent role [15]. On one hand, it can suppress tumor progression by inducing ICD, which activates dendritic cells and enhances T-cell-mediated antitumor immunity [16]. On the other hand, necroptosis may promote tumor progression by fostering an immunosuppressive and pro-metastatic microenvironment through the release of proinflammatory cytokines [17]. The overall biological outcome likely reflects the balance between these opposing effects. Notably, the expression levels of necroptosis-related proteins (e.g., RIPK1, RIPK3, MLKL) are frequently altered in cancers, often through epigenetic silencing, and these alterations are correlated with patient prognosis and treatment response [18], highlighting their potential as therapeutic targets for strategic induction.

Collectively, necroptosis represents a stress-unmasked cell death program that becomes dominant when apoptotic checkpoints are compromised, providing a conceptual framework for understanding its context-dependent roles in cancer. While several recent reviews have described necroptosis in tumors, a mechanistically integrated perspective linking molecular pathways, dual functional outcomes, and emerging necroptosis-inducing strategies is still lacking. Here, we provide such an integrative overview, highlighting how stress-amplified necroptotic signaling can be leveraged across small molecules, natural products, and nanomedicine platforms, and discussing the therapeutic implications, current challenges, and future directions for translating necroptosis into effective anticancer interventions.

## Mechanism of necroptosis

Considering the significance of necroptosis in cancer, a thorough understanding of its mechanisms is essential for developing novel strategies to modulate necroptosis in cancer therapy. As a PCD, many factors played important roles in regulating necroptosis (Table 1). The current understanding of necroptosis mechanisms primarily comes from studies on the TNF-α signaling pathway [19]. TNF-α, a key cytokine, mediates the inflammatory response and initiates cellular activities [20]. When TNF-α binds to TNFR1 on the cell membrane, the resulting signaling pathway primarily facilitates cellular growth, survival, and the induction of inflammatory responses. However, under specific conditions, TNF-α can also trigger PCD. Recent studies have classified TNF-α-induced PCD into two distinct types: apoptosis and necroptosis.

**Table 1:** Key factors and their function in necroptosis.

Key factor	Function in necroptosis	Inhibitors	References
TNF-α	Mediating classical necroptosis; forming complex I	Infliximab; Etanercept	[126–129]
cIAP1/2	Polyubiquitinating RIPK1 to induce NF-κB signaling	Smac mimetics	[130, 131]
CYLD	Deubiquitinating RIPK1; Promoting the activation of RIPK1 in the necrosome	-	[132, 133]
Caspase-8	Suppressing necroptosis; Cleaving RIPK1 and RIPK3 and activating apoptosis; Cleaving CYLD to promote cell survival	zVAD-FMK	[134–136]
RIPK1	Determining the survival or death of cell; Recruiting and activating RIPK3 to form necrosome	Necrostatin-1	[7, 137]
RIPK3	Participating in the formation of necrosome; Phosphorylating MLKL	GSK-843; GSK-872	[138, 139]
MLKL	Oligomerized and translocated to plasma membrane to execute necroptosis	Necrosulfonamide; TC13172; GW806742X	[140–142]

## Regulation of necroptosis signaling by post-translational modifications

**Fig. 1:**
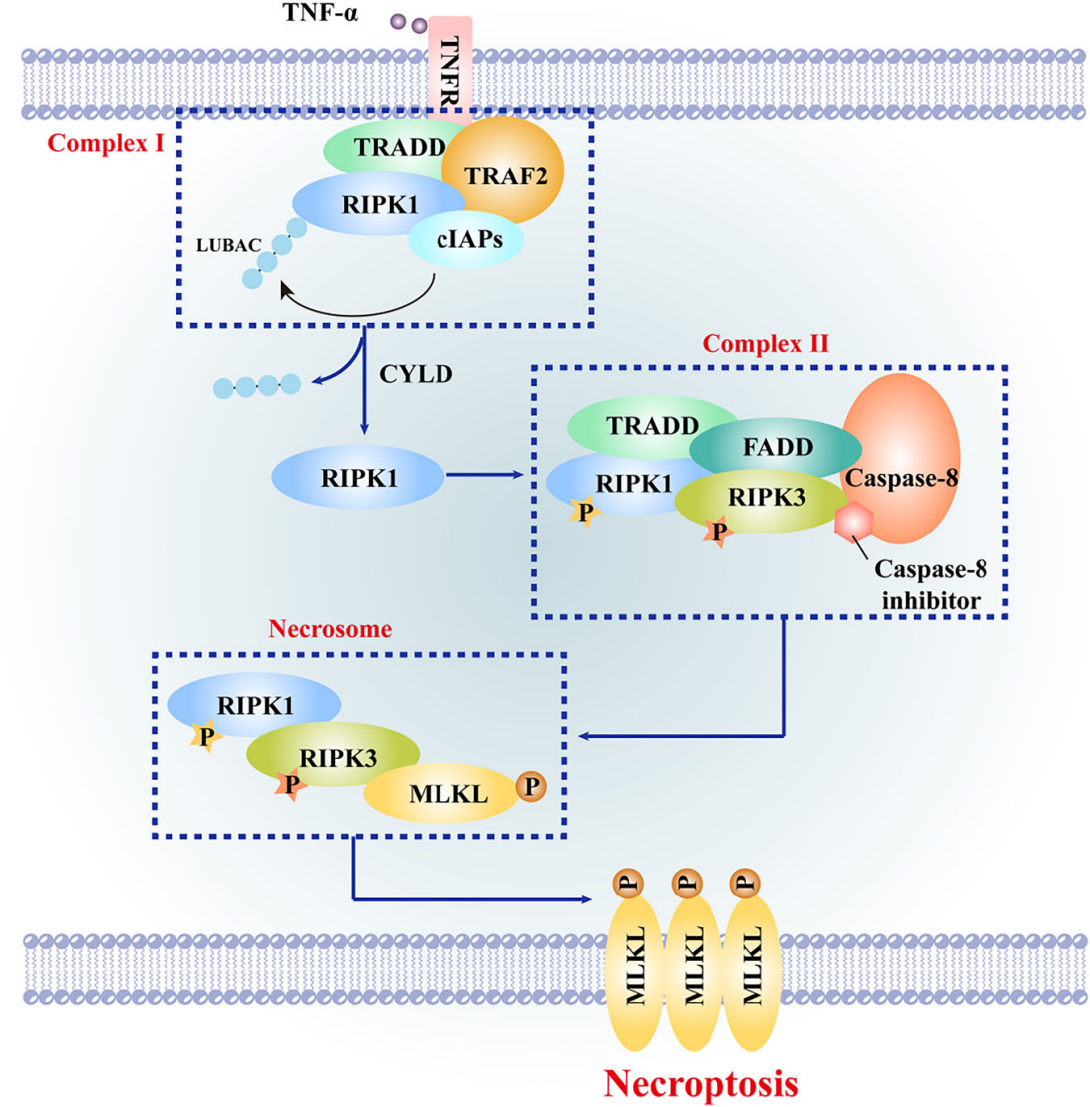
Schematic overview of the TNFR1-mediated necroptosis signaling pathway. TNF-α binding to TNFR1 triggers the rapid recruitment of RIPK1 and assembly of Complex I at the receptor, which includes TRADD, TRAF2, cIAPs, and potentially LUBAC (leading to CYLD involvement). Under conditions where caspase-8 activity is inhibited (e.g., by pharmacological or viral caspase-8 inhibitors), Complex I transitions to Complex II, incorporating FADD, RIPK1, RIPK3, and caspase-8. In the absence of active caspase-8, RIPK1 and RIPK3 become phosphorylated (P), enabling formation of the necrosome. Within the necrosome, activated RIPK3 phosphorylates MLKL, leading to MLKL oligomerization (shown as phosphorylated multimers), plasma membrane translocation, and execution of necroptosis. When caspase-8 is active, it cleaves and inactivates RIPK1 and RIPK3, preventing necroptosis.

The necroptotic pathway is primarily regulated through post-translational modifications (PTMs) of key signaling proteins, which function as molecular switches to encode cell fate decisions downstream of death receptor signaling [21]. The molecular mechanism of necroptosis was initially characterized following activation of the TNFR1 (Fig. 1). Upon receptor stimulation, RIPK1 is rapidly recruited to the receptor complex, assembling into a supramolecular signaling platform termed complex I [22, 23]. This complex comprises multiple regulatory proteins, including TNFR1-associated death domain protein (TRADD) [24, 25], cellular inhibitor of apoptosis proteins 1/2 (cIAP1/2) [26], and TNF receptor-associated factor 2 (TRAF2) [27].

**Fig. 2:**
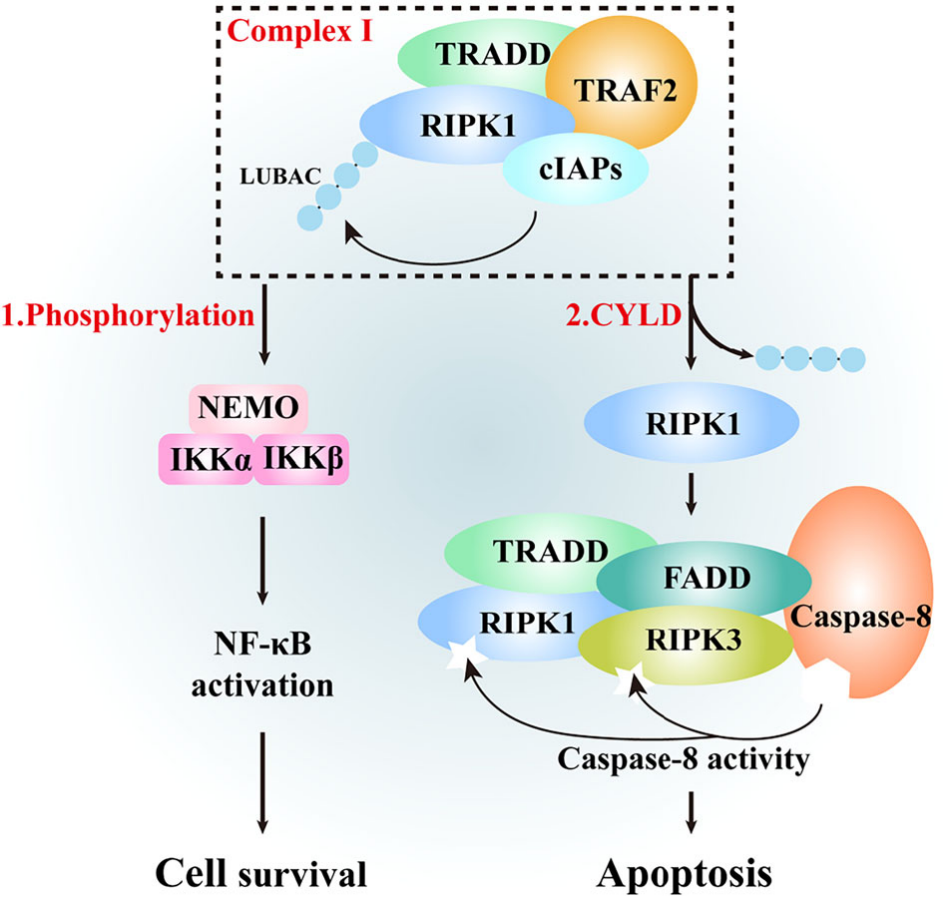
Schematic representation of RIPK1 regulation in TNFR1 signaling complexes controlling cell fate. In complex I, RIPK1 is recruited with TRADD, TRAF2, cIAPs, and LUBAC, and undergoes phosphorylation (by IKKα/β and NEMO) and ubiquitination. These PTMs promote NF-κB activation for cell survival while restraining RIPK1 cytotoxicity. Deubiquitination by CYLD allows RIPK1 to dissociate and form cytosolic complex II with TRADD, FADD, RIPK3, and caspase-8, leading to either NF-κB-mediated survival or caspase-8-dependent apoptosis.

Within complex I, RIPK1 is subject to two critical PTMs: phosphorylation, mediated by kinases such as IκB kinase β (IKKβ), which modifies specific serine/threonine residues [28, 29]; and ubiquitination, catalyzed by E3 ligases including cIAP1/2, which conjugate polyubiquitin chains to RIPK1 [30, 31]. These modifications serve dual regulatory functions by promoting nuclear factor-κB (NF-κB) signaling to support cell survival and inflammatory responses, while simultaneously retaining RIPK1 within complex I to restrain its cytotoxic potential [32]. Failure to establish or maintain these PTMs permits RIPK1 to disengage from complex I, thereby enabling its transition into a secondary cytosolic signaling hub, complex II, which represents a critical decision node in regulated cell death signaling (Fig. 2).

Importantly, the formation of complex II does not inevitably culminate in necroptosis, as caspase-8-mediated proteolytic cleavage of RIPK1 and RIPK3 constitutes a critical checkpoint that suppresses programmed necrosis [33]. Consequently, necroptosis execution requires the concurrent satisfaction of two conditions: inhibition of caspase-8 activity, achieved experimentally via pharmacological inhibitors, genetic ablation, or viral-encoded suppressors during infection; and elevated expression of cellular FLICE-inhibitory protein (cFLIPS) isoforms [34–36]. Under these conditions, RIPK1 accumulates within complex II and facilitates RIPK3 recruitment and activation through reciprocal interactions between their RIP homotypic interaction motifs (RHIMs), ultimately leading to RIPK3-mediated phosphorylation of MLKL, the terminal effector of necroptotic cell death [37–39].

Collectively, these findings establish caspase-8 as a central molecular checkpoint that governs the bifurcation of complex II signaling toward apoptotic or necroptotic outcomes. Preservation of caspase-8 activity favors apoptotic execution, whereas its inhibition or loss licenses RIPK1/RIPK3/MLKL-driven necroptosis, underscoring the tightly coupled and context-dependent nature of these two regulated cell death pathways.

### Other mechanism of necroptosis

**Fig. 3:**
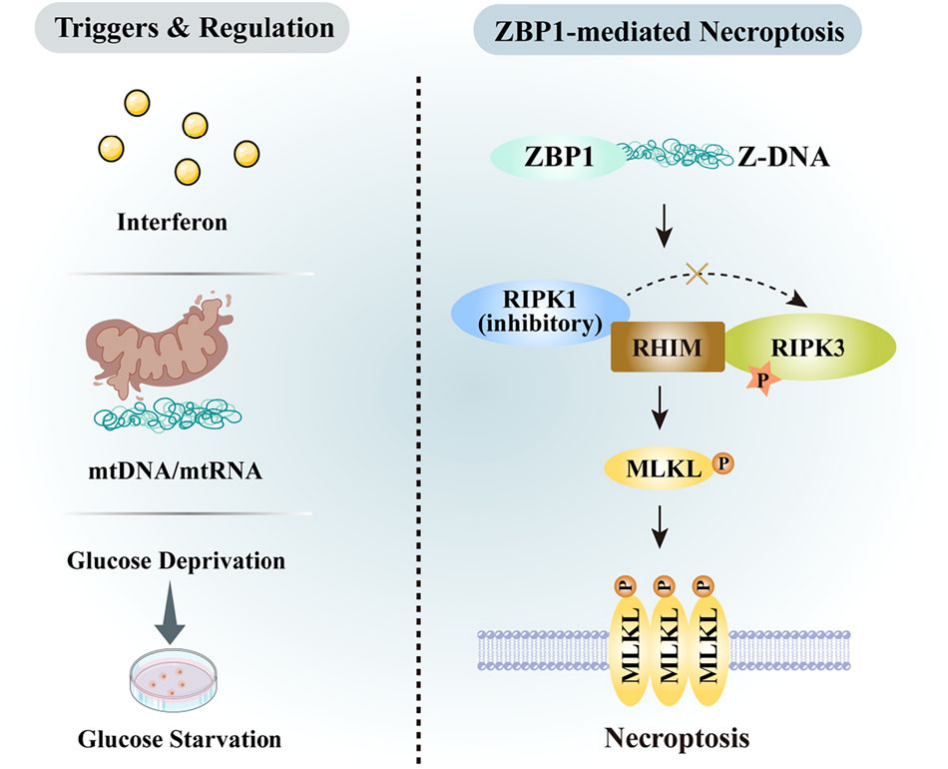
ZBP1-mediated necroptosis pathway. ZBP1 senses Z-DNA (e.g., from mtDNA released under glucose starvation or other stress) and directly engages RIPK3 via RHIM domains, leading to RIPK3 phosphorylation and subsequent MLKL activation and necroptosis. RIPK1 can inhibit this interaction. Upstream triggers include interferon-induced ZBP1 upregulation and metabolic stress (glucose deprivation) that promotes mtDNA release.

In addition to the canonical necroptotic pathways, ZBP1 has emerged as an important sensor and mediator of necroptosis in a context-dependent manner. ZBP1 directly interacts with RIPK3 through RHIM-domain interactions, leading to RIPK3 autophosphorylation and subsequent MLKL activation, ultimately resulting in membrane disruption and necroptotic cell death [40]. Importantly, ZBP1-mediated necroptosis can occur independently of RIPK1 in some contexts, as the presence of RIPK1 can competitively inhibit the ZBP1-RIPK3 interaction via RHIM motifs, dampening ZBP1 signaling under basal conditions [41, 42]. Accumulating evidence indicates that the role of ZBP1 in cancer is highly context specific. In preclinical breast cancer models, ZBP1 expression is dramatically elevated in necrotic tumor regions, and deletion of ZBP1 markedly blocks tumor necroptosis and inhibits metastasis, indicating that metabolic stress such as glucose deprivation triggers ZBP1-dependent necroptosis via mtDNA release and binding to ZBP1 to activate RIPK3/MLKL signaling [40, 43]. In contrast, in some tumor types ZBP1 expression and downstream necroptotic components are suppressed, suggesting selective pressure against ZBP1-mediated inflammatory cell death during tumor progression. The activity of ZBP1 is also regulated by innate immune signaling. Interferon treatment has been shown to upregulate ZBP1 expression and drive necroptosis in the absence of RIPK1, linking innate antiviral pathways to necroptotic cell death *via* ZBP1-RIPK3 complexes and highlighting complex regulatory layers involving interferon signaling and stress responses [44, 45]. Collectively, these findings highlight ZBP1 as a context-dependent regulator of necroptosis whose therapeutic relevance in cancer is determined by the balance between immune activation and inflammation-driven tumor progression (Fig. 3).

## The context-dependent dual role of necroptosis in tumor biology

**Fig. 4:**
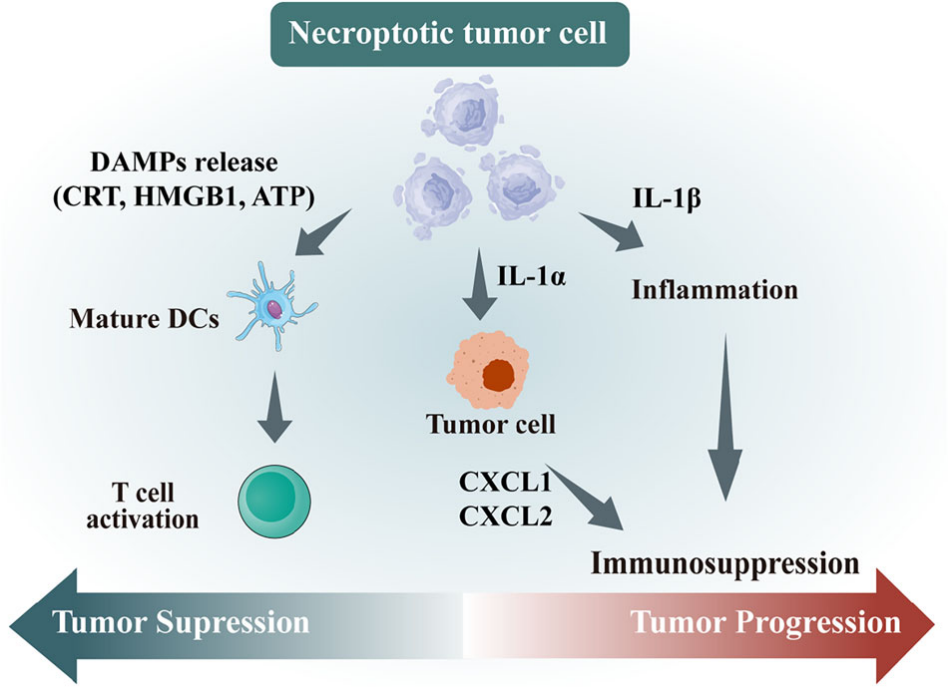
The context-dependent dual role of necroptosis in tumor biology. This figure illustrates how necroptosis in tumor cells can exert either tumor-suppressive or tumor-promoting effects. Necroptosis triggers the release of DAMPs such as CRT, HMGB1, and ATP, which activate dendritic cells and cytotoxic T lymphocytes, thereby enhancing antitumor immunity. Conversely, the release of cytokines like IL-1α, IL-1β, and chemokines (e.g., CXCL1, CXCL2) can induce inflammation and immune suppression, thereby facilitating tumor progression. The overall outcome is determined by the specific tumor microenvironment and immune context.

Necroptosis is increasingly recognized as a context-dependent regulator of tumor biology rather than a uniformly tumor-suppressive or tumor-promoting process. Its biological consequences are determined by tumor-intrinsic signaling states, the cellular origin of necroptotic death, and the immune architecture of the tumor microenvironment (TME). As an inflammatory form of regulated cell death, necroptosis links cell death execution to immune modulation, thereby positioning it at the intersection of tumor control and inflammation-driven tumor progression [46, 47] (Fig. 4).

A key determinant of necroptosis-associated outcomes is the cellular compartment in which necroptotic signaling is activated. Necroptosis in tumor cells often exerts tumor-suppressive effects by eliminating malignant cells while promoting ICD [48, 49]. Activation of the RIPK1/RIPK3/MLKL axis leads to membrane permeabilization and the release of damage-associated molecular patterns (DAMPs), including CRT, HMGB1, and ATP, which facilitate dendritic cell (DC) maturation and cytotoxic T lymphocyte (CTL) priming [50, 51].

Beyond cell death itself, the qualitative profile of cytokines and chemokines released during necroptosis critically shapes immune outcomes. Necroptosis-associated release of IL-1 family cytokines, TNF-α, and chemokines such as CXCL1 and CCL2 can either reinforce antitumor immunity or foster chronic inflammation [17, 47, 52]. While IL-1β is often linked to DC activation and Th1-polarized immune responses, IL-1α and sustained chemokine production may promote myeloid-derived suppressor cell (MDSC) recruitment, angiogenesis, and immune evasion. Thus, the immune consequences of necroptosis reflect a balance between immunostimulatory and immunosuppressive inflammatory circuits.

Clinical correlations between necroptosis-related factors and patient prognosis further highlight this context dependency (Table 2). Reduced expression of RIPK3 or MLKL is associated with poor outcomes in several cancer types, consistent with impaired ICD and immune surveillance [53–61]. Conversely, elevated necroptotic signaling has been linked to tumor progression in others, likely reflecting differences in immune contexture and microenvironmental wiring [46, 62–65]. Collectively, these observations support a model in which necroptosis functions as a tunable determinant of tumor fate, governed by cellular identity and TME-specific inflammatory programs, rather than an intrinsically beneficial or detrimental process.

**Table 2:** Effects of key necroptosis factors on tumor prognosis.

Cancer type	Necroptosis factors	Cancer prognosis	References
Colorectal cancer	Low expression of RIPK3 and MLKL	Promoted tumor development and reduced RFS and OS	[55, 56, 62]
Gastric cancer	Low expression of MLKL	Poor prognosis and reduced OS	[60]
Breast cancer	Low expression of RIPK3	Poor prognosis	[54]
Acute myeloid leukemia	Low expression of RIPK3 and MLKL	Promoted tumor development	[57, 143]
Melanoma	Absence of RIPK3	-	[58]
Ovarian cancer	Low expression of RIPK3 and MLKL	Poor prognosis and reduced OS	[59, 144]
Lung cancer	High expression of RIPK1	Promoted tumor development	[63]
Pancreatic cancer	High expression of RIPK1, RIPK3, and MLKL	Promoted tumor development	[46, 61, 65]
Glioblastoma	High expression of RIPK1	Poor prognosis	[64]

## Context-dependent features of necroptosis compared with other cell death pathways

Compared with other regulated cell death modalities, necroptosis exhibits several context-dependent features that distinguish it as both an attractive and potentially risky therapeutic strategy in cancer (Table 3). Unlike apoptosis, which is often disabled in advanced tumors due to defects in caspase activation or p53 signaling, necroptosis bypasses caspase dependence and can therefore eliminate apoptosis-resistant cancer cells through RIPK1/RIPK3/MLKL-mediated membrane disruption [66]. However, in contrast to apoptosis, necroptosis is inherently pro-inflammatory due to plasma membrane rupture and the release of DAMPs [51]. While this feature may enhance antitumor immunity by promoting dendritic cell activation and T-cell priming, it also differentiates necroptosis from ferroptosis and pyroptosis in terms of inflammatory magnitude and outcome [67, 68]. Pyroptosis is typically associated with rapid cytokine release and acute inflammation, whereas ferroptosis exhibits a more context-dependent immunogenic profile linked to lipid peroxidation. In this regard, necroptosis occupies an intermediate position, where insufficient control may shift its role from immune activation toward tumor-promoting inflammation. Therefore, although necroptosis offers a unique opportunity to overcome apoptosis resistance, its therapeutic application requires precise spatiotemporal regulation to avoid excessive inflammation and collateral tissue damage.

**Table 3:** Context-dependent features of necroptosis compared with other regulated cell death pathways in cancer.

Cell death modality	Key executors	Inflammatory potential	Therapeutic limitation
Apoptosis	Caspase-3/7	Low	Frequent resistance in tumors
Necroptosis	RIPK1/RIPK3/MLKL	Moderate-high	Risk of excessive inflammation
Pyroptosis	Gasdermins	High	Systemic cytokine toxicity
Ferroptosis	Lipid peroxidation	Context-dependent	Tissue-specific toxicity

## Inducers of necroptosis in cancer therapy

### Small molecule agents

The induction of necroptosis in tumor cells represents a promising therapeutic strategy in oncology, as it can circumvent apoptosis resistance and enhance antitumor immunity. Accumulating evidence indicates that a broad spectrum of small molecules and natural products can trigger necroptosis through distinct molecular mechanisms (Table 4). For clarity, these agents can be broadly categorized into conventional chemotherapeutics, kinase inhibitors and metal-based compounds, and natural products.

1. Caspase/proteostasis-inhibition-facilitated necroptosis induced by conventional anticancer agents: Several conventional anticancer agents induce necroptosis not by directly activating necroptotic kinases, but by imposing cellular stress while simultaneously disabling apoptotic checkpoints, thereby unmasking RIPK1-dependent necroptotic signaling. For example, 5-fluorouracil in combination with the pan-caspase inhibitor Z-VAD induces necroptosis in colorectal cancer *via* RIPK1/NF-κB signaling [69]. In addition, proteasome inhibitors such as bortezomib/DOX and MG132 promote RIPK3-MLKL-dependent necroptosis in cancer cells [70, 71].
2. Kinase inhibitors and metal-based synthetic compounds: Targeted kinase inhibitors and metal-based compounds can induce necroptosis indirectly by imposing severe cellular stress rather than by directly engaging necroptotic kinases. The PLK1 inhibitor BL2536 induces mitotic catastrophe-associated necroptosis in prostate cancer, whereas Compound C, an AMPK inhibitor, triggers necroptosis in glioma cells through metabolic stress. Similarly, the ruthenium(II) complex Ru7, which functions as a dual topoisomerase inhibitor, elicits genotoxic stress and activates the canonical RIPK1/RIPK3/MLKL signaling cascade [72–74].

**Table 4:** Small-molecule drug that induce necroptosis in cancer therapy.

Name	Cancer	Mechanism	References
5-Fluorouracil+Z-VAD	Colorectal cancer	Inducing TNFα-dependent necroptosis driven by RIPK1 kinase and NF-κB	[69]
MG132 + DOX	Lung cancer	RIPK3-dependent necroptosis	[70]
MG132+bortezomib	Leukemia	RIPK3-MLKL necroptotic pathway	[71]
BI2536	Prostate cancer	Leading to mitotic catastrophe	[72]
Compound C	Glioma	Calpain/Cathepsin-mediated	[73]
Ruthenium (II) complexes	Lung cancer	Via the RIPK1/RIPK3/MLKL pathway	[74]
Shikonin	Glioma; Leukemia; Breast cancer; Osteosarcoma; Prostate cancer	ROS overproduction; Promoting RIPK1/RIPK3 necrosome formation	[75, 77–80]
Staurosporine	Lymphoma	Inducing RIPK1 and MLKL-dependent necroptosis	[81]
Matrine	Cholangiocarcinoma	Upregulating expression of RIPK3 and plasma membrane translocation of MLKL	[82]
Celastrol	Gastric cancer	Promoting the translation of MLKL	[83]
Resibufogenin	Colorectal cancer	Upregulating expression of RIPK3	[84]
Tanshinone IIA	Liver cancer	Promoting RIPK1/RIPK3 necrosome formation	[85]
Emodin	Glioma	Via the TNF-α/RIPK1/RIPK3 pathway	[86]
Ganoderic acid T	Cervical cancer	ROS generation	[87]
Artesunate	Schwannoma	Activating RIPK1	[88]
Bufalin	Breast cancer	ROS production and upregulating RIPK1/RIPK3/PGAM5 pathway	[89]
Ophiopogonin D'	Prostate cancer	Activating RIPK1	[90]

(3) Natural products and phytochemicals: Natural products and phytochemicals predominantly induce necroptosis by converging on stress-amplified RIPK signaling and necrosome assembly, rather than acting as direct kinase agonizts. Most compounds elevate intracellular ROS or disrupt redox homeostasis, lowering the activation threshold of the RIPK1/RIPK3/MLKL axis. Shikonin serves as a prototypical example, inducing ROS accumulation, upregulating RIPK proteins, promoting necrosome formation, and overcoming chemoresistance in various cancers [75–80]. Beyond shikonin, compounds like staurosporine and matrine modulate RIPK/MLKL translocation in specific cancers [81, 82]. Other phytochemicals, including celastrol [83], resibufogenin [84], tanshinone IIA [85], emodin [86], ganoderic acid T [87], artesunate [88], bufalin [89], and ophiopogonin D [90], also induce necroptosis through diverse upstream stress signals, highlighting the versatility of natural products in activating this cell death pathway.

Together, these studies underscore the mechanistic diversity of small-molecule necroptosis inducers and highlight that their therapeutic efficacy is tightly constrained by tumor-specific signaling states, including caspase competency, redox balance, and RIPK3 expression, emphasizing the need for context-informed application of necroptosis-based strategies.

### Nanomedicine-induced necroptosis

**Fig. 5:**
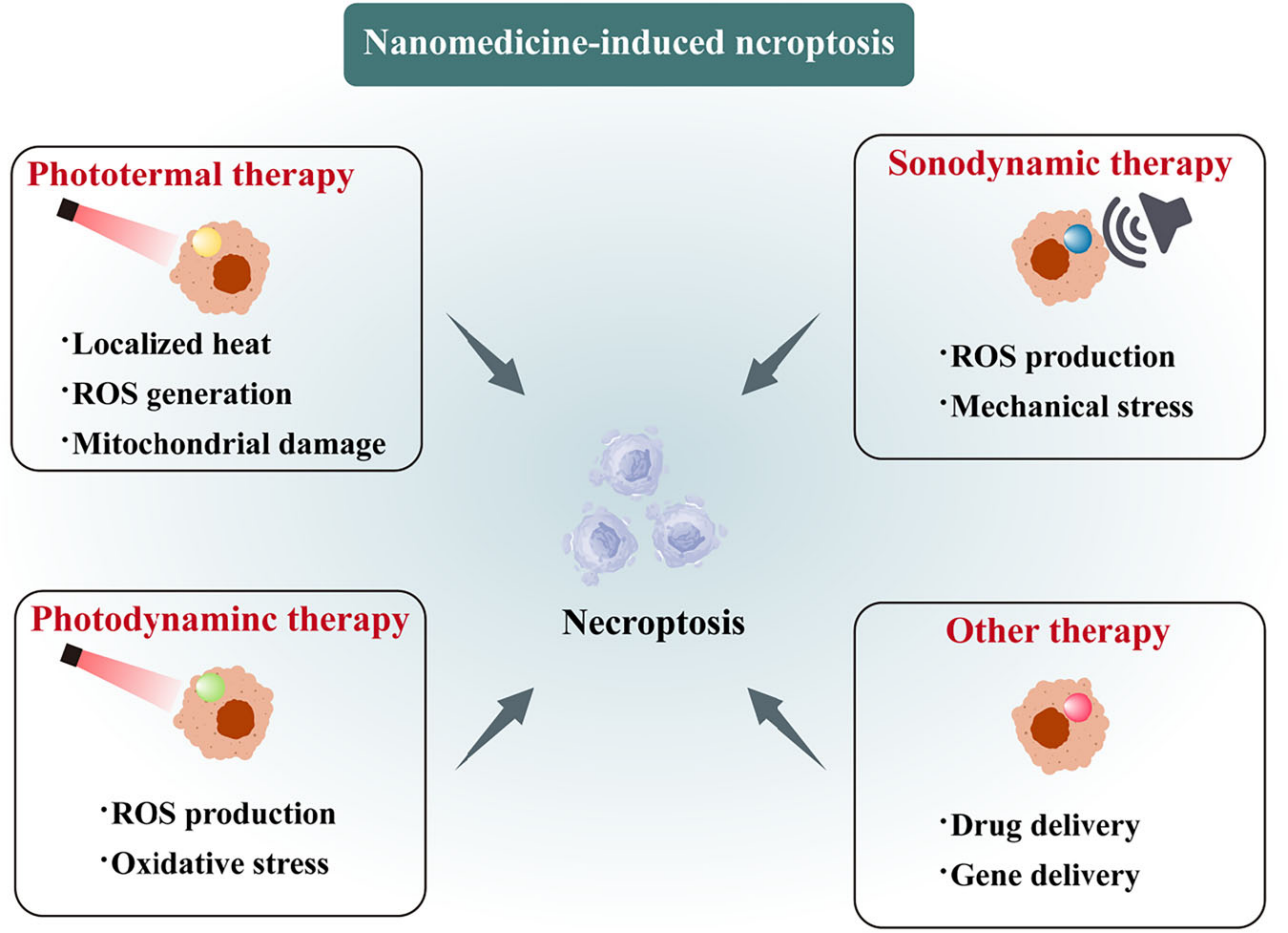
Nanomedicine-induced necroptosis. Key strategies include: photothermal therapy, photodynamic therapy, sonodynamic therapy and other therapies.

Nanomedicine-based strategies provide a tumor-selective platform for inducing necroptosis by integrating the preferential tumor accumulation enabled by the enhanced permeability and retention (EPR) effect with spatiotemporally controllable activation mechanisms [91]. The activation is typically confined to tumor sites using external stimuli or tumor-specific intracellular cues such as acidic pH, hypoxia, and redox imbalance [92]. Importantly, tumor cells often exhibit impaired apoptotic signaling and heightened sensitivity to oxidative and metabolic stress, making them more susceptible to RIPK1/RIPK3/MLKL-mediated necroptosis than normal tissues. Together, these layered mechanisms of selectivity reduce off-target toxicity while enabling robust necroptosis activation within the tumor microenvironment (Fig. 5).

### Photothermal therapy (PTT)

PTT-based nanomedicines exploit localized heat generation under near-infrared (NIR) irradiation to induce membrane disruption, mitochondrial dysfunction, and oxidative stress, providing a spatially and temporally controllable trigger for necroptosis activation. Increasing evidence suggests that PTT can synergize with necroptosis to enhance antitumor immunity [93, 94]. For example, iRGD-modified gold nanocages combined with epigallocatechin gallate can induce a time-dependent shift from necroptosis to apoptosis while enhancing therapeutic efficacy through HIF-1α downregulation [95]. Similarly, BiVO4/MoS2@HA nanoparticles leverage a p-n heterojunction to amplify photothermal effects and ROS generation, thereby overcoming hypoxia-associated limitations in PTT and activating necroptotic pathway [96]. Additionally, siCDK7-loaded gold nanoparticles promote tumor necroptosis, remodel the immunosuppressive tumor microenvironment, and enhance the therapeutic efficacy of PD-1 blockade [97]. Other nanomaterials, including carbon nanodots and MoS2 nanoflowers, and black phosphorus nanosheets, further demonstrate that optimized photothermal parameters can activate necroptosis, enhance ICD, and improve photo-immunotherapeutic outcomes [98–100].

### Photodynamic therapy (PDT)

Nanomaterial-enabled PDT induces necroptosis primarily through ROS-mediated damage, while advanced designs aim to overcome tumor hypoxia and enhance ICD. Oxygen-independent or organelle-targeted PDT systems have been developed to enhance necroptosis induction in resistant tumors. For instance, aryl-ketone substituted cyanine (ACy-5F) acts as an oxygen-independent photoinitiator that generates alkyl radicals under red light, triggering necroptosis in hypoxic tumors [101]. Methylene blue (MB)-PDT induces necroptosis in prostate and pancreatic cancer cells through oxidative stress, lysosomal disruption, and crosstalk with autophagy, with RIPK1, RIPK3, and MLKL serving as key mediators of therapeutic efficacy [102, 103]. In breast cancer, MB-PDT selectively exploits polyunsaturated fatty acid-rich membranes to provoke lysosome-dependent necroptosis [104]. Additionally, 5-aminolevulinic acid-PDT promotes ICD and enhances antitumor immunity [105]. Nanomaterials like nitrogen-doped TiO₂ nanoparticles enable light-controllable switching from autophagy to necroptosis in melanoma [106]. Moreover, mitochondria-targeting agents including self-assemble TPA-N-8 nanospheres, generate ROS in a light-independent manner to induce PINK1-mediated mitophagy and DNA damage-driven necroptosis [107]. A biomimetic nanosystem (R-CM@MSN@BC) that integrates PDT with glutamine metabolic therapy has also been shown to remodel the tumor microenvironment and enhance immunotherapy efficacy [108].

### Sonodynamic therapy (SDT)

SDT employs low-intensity ultrasound to activate sonosensitizers accumulated within deep tumor tissues, leading to ROS generation and mechanical stress that can initiate necroptosis and subsequent immune activation [109, 110]. Compared with light-based approaches, SDT offers superior tissue penetration and non-invasive control. Recent advances in sonosensitizer design and delivery systems have significantly improved SDT efficacy and its immunomodulatory potential [111]. For instance, an ultrasound-responsive nano-microbubble system induces necroptosis, promotes plasma membrane disintegration, and facilitates DAMPs release, thereby enhancing dendritic cell maturation and CD8^+^ T cell activation [112]. In addition, a membrane-anchored iridium(III) complex-based nanosonosensitizer induces multiple modes of regulated cell death, including necroptosis, pyroptosis, and apoptosis, resulting in amplified immune responses [113]. Nanovesicles encapsulating indocyanine green (ICG) combined with shikonin further demonstrate that SDT can induce necroptosis in liver cancer cells while suppressing invasion and metastasis [114]. Piezoelectric CBNO-OV1 nanosheets, by generating ROS and Ca²⁺ influx under ultrasound, effectively induce necroptosis and trigger a self-reinforcing antitumor immune cycle [115].

### Other treatments

Beyond stimulus-responsive therapies, certain nanomedicines directly induce necroptosis by targeting key signaling pathways without the need for external activation. Among these approaches, targeted gene delivery represents a powerful strategy to selectively engage necroptotic machinery. For instance, a highly branched poly (β-amino ester)-based delivery system was developed deliver MLKL plasmid DNA to tumor sites, thereby inducing necroptosis, enhancing antitumor immune response, and suppressing metastasis [116]. Similarly, silica nanoparticles delivering RIPK3 mRNA effectively triggered necroptosis in liver cancer cells and promoted immune cell infiltration [117]. Targeted delivery of small-molecule drugs has also garnered significant attention. An aptamer-targeted Prussian blue nanoparticle co-delivery of shikonin and silver nanoparticles activated the RIPK3/MLKL pathway, elicited ICD, and enhances CD8⁺ and CD4⁺ T cell infiltration in TNBC [50]. Additional shikonin-containing nanocomplexes further support the role in anti-tumor immunity [118–120]. Furthermore, a dendrimer-functionalized metal-phenolic nanomedicine encapsulating maleimide-modified doxorubicin induced necroptosis, suppressed both oxidative phosphorylation and glycolysis, and significantly inhibited tumor growth and peritoneal metastasis in colorectal cancer models [121]. Other metallic nanoplatforms, including Mg²⁺-doped hydroxyapatite nanoparticles and supra-structured cryo-nanocatalysts, further demonstrate that nanomedicine-enabled necroptosis can overcome apoptosis resistance and synergize with immune checkpoint blockade [122].

Collectively, nanomedicine-based strategies enable precise spatial and temporal control of necroptosis induction across diverse therapeutic modalities. Although these approaches exhibit promising antitumor and immunomodulatory effects, most studies remain confined to preclinical models, underscoring the need for further evaluation of tumor selectivity, biosafety, and translational potential.

## Conclusion and future perspectives

This article discusses the advancements in necroptosis research in the context of tumor treatment, including its mechanisms, relationship with tumor therapy, and the various inducers. Tumor cell necroptosis can be initiated through multiple signaling pathways, with RIPK1, RIPK3, and MLKL playing central roles, ultimately causing cell membrane rupture and the release of cytokines. Necroptosis influences cancer progression, metastasis, and immune surveillance, and patient prognosis, making its induction a promising therapeutic strategy. Necroptotic cells have the can elicit both innate and adaptive immune responses, enhancing anti-tumor immunity. Various agents, including natural compounds and nanomedicines, have been identified as necroptosis inducers, providing opportunities for novel therapeutic approaches.

Despite its potential, several challenges must be addressed to enable clinical translation. Firstly, reliable biomarkers for the clinical identification of necroptosis are limited. Phosphorylated RIPK3 and MLKL are commonly used to assess activity [123], but they can also occur in pyroptosis [124], necessitating multifactorial validation, such as MLKL membrane translocation. Secondly, most tumors exhibit low RIPK3 or MLKL expression, often due to hypermethylation of the RIPK3 gene. Agents like decitabine can restore RIPK3 levels and sensitize tumors to chemotherapy [54], though further clinical evaluation is needed. Thirdly, necroptosis is an inflammatory response that has the potential to facilitate tumor growth. The recruition of inflammatory cells during necroptosis may contribute to tumor development by enhancing angiogenesis, stimulating cancer cell proliferation, and accelerating the process of cancer metastasis [125]. Finally, Once the induction of necroptosis has been established, it is imperative to explore how this method can be integrated with clinical treatment protocols. The development of novel clinical treatment strategies should involve the combination of these approaches with existing therapeutic modalities [21]. It is essential to identify which of the current standard treatments are most likely to synergize with necroptosis induction regimens, mitigate the inflammatory responses they may provoke, enhance therapeutic efficacy, reduce toxicity, and ultimately provide a safer treatment option for cancer patients. This integration is crucial for advancing the clinical application of necroptosis.

Overall, induced necroptosis holds significant promise as a cancer therapy, but its clinical application requires careful consideration of mechanistic constraints, potential risks, and strategy for integration with standard treatments. This review provides an integrative perspective linking molecular mechanisms, dual functional outcomes, and emerging therapeutic strategies, offering a framework to guide future research and the safe translation of necroptosis-based interventions.
